# A Pair of Testicular Torsion Medicolegal Cases with Caveats: The Ball’s in Your Court

**DOI:** 10.5811/cpcem.2018.10.39497

**Published:** 2018-10-18

**Authors:** John B. Bass, Kyle S. Couperus, Jamie L. Pfaff, Gregory P. Moore

**Affiliations:** * Madigan Army Medical Center, Department of Emergency Medicine, Tacoma, Washington; † University of Texas Southwestern School of Medicine, Dallas, Texas

## Abstract

In this article, we present two medicolegal cases illustrating medical and diagnostic pitfalls that can lead to litigation for missed testicular torsion. Testicular torsion (TT) is a urologic emergency with potentially devastating consequences and costs, for providers and patients alike. TT occurs in approximately 4.5 per 100,000 males under the age of 25. While uncommon, TT is the third most common cause of medical malpractice suits in this demographic. As a consequence of varying presentations and physical exam findings, and diagnostic imaging subject to individual interpretation, this time-sensitive diagnosis may be missed by emergency department providers. Delays in diagnosis significantly increases the morbidity associated with TT, and 31.9%–41.9% of such cases result in testicular loss. The average reported settlement for TT malpractice litigation is $60,000. This article discusses two actual malpractice cases involving TT and provides insight and caveats to ensure an optimal evaluation and diagnostic approach to this often-elusive condition.

## CASE 1: Anonymous v. Anonymous

A 16-year-old male arrived at the emergency department (ED) complaining of right lower quadrant abdominal pain with some associated nausea and vomiting. The emergency physician (EP) completed an abdominal exam, obtained labs, an abdominal ultrasound, and a computed tomography (CT) of the abdomen and pelvis. These were all unremarkable. Nevertheless, a surgical consultation was obtained to further evaluate for appendicitis. The surgeon did not feel appendicitis was present, and the patient was discharged. A genital exam was never performed. The following day, the patient returned with right testicular pain. He was immediately taken to the operating room for scrotal exploration and required a right orchiectomy. A lawsuit was initiated for failure to perform a genital exam, and failure to consider testicular torsion (TT) in the diagnosis. Before trial a settlement of $300,000 was reached.^3^ Isolated abdominal pain is a frequent chief complaint associated with TT, and one review found that failure to complete a testicular exam was associated with 19% of TT malpractice cases.^2^ It is imperative to consider this diagnosis whenever lower abdominal pain is present and complete a scrotal exam.

## CASE 2: Graham v. Noreldin

A 14-year-old male was taken to the ED after awakening with abdominal pain. Laboratory studies, an abdominal CT, and a scrotal ultrasound were done. The CT was read as suggestive of appendicitis and thus a surgical consultation was obtained. The surgeon did not feel that appendicitis was present. The radiologist reviewed the ultrasound and diagnosed epididymitis. Based on the studies the EP discharged the patient on antibiotics. Three days later the patient awoke with testicle pain and was taken to a different ED where he was diagnosed with TT and received an orchiectomy. A review of the original ultrasound revealed there was decreased blood flow to the testicle. The patient litigated claiming that the diagnosis should have been made on the first visit and the testicle could have been salvaged. The case was solely against the EP and not the radiologist. There was testimony from the EP that he had ordered the “gold standard” test and relied on the interpretation by radiology. After trial, the jury awarded a $500,000 verdict.^4^ This case is typical of others. When a radiologist misreads the testicular ultrasound, often the radiologist pays out less than the EP, or the EP pays out alone. The thought process was that the EP had the ability to make a “clinical correlation” that the radiologist could not make.

## DISCUSSION

### Dr. Bass

There is no standard presentation for TT. Testicular torsion presentation can present similarly to epididymitis. A significant number of proven TT cases present with gradual onset discomfort, whereas alternative causes of scrotal pain, such as epididymitis, can present with sudden discomfort in up to 51% of cases.^1^ Finally, circumstances surrounding the presentation may not reveal the ultimate diagnosis. TT is attributed to direct trauma in 4–8% of reported cases, and more frequently occurs during sleep, as a result of spontaneous cremasteric contractions.^5^ Since there is a wide variety and overlap of symptoms and circumstances surrounding TT, it is imperative to not rely on historical features alone to guide further evaluation.

EPs should be hesitant to decide the absence (or presence) of TT based solely on clinical exam. Presence or absence of cremasteric reflexes, scrotal edema/erythema, pain along the upper pole of the testicle or epididymis, enlarged epididymis, transverse lie, Prehn’s sign (pain relief with examiner lifting testicle), and retraction of testicle all fail to give a definitive answer.^1^ Even when experienced urologists combine all these exam findings their initial impressions are frequently in error.^1^

The presence of a cremasteric reflex has historically been touted to rule out TT. This unfortunately is not completely true. Several case series, although mostly small, have reported TT with intact cremasteric reflexes.^1^ Specifically, patients who were later diagnosed with TT had intact cremasteric reflexes in 12%–40% of cases.^1^ Cremasteric reflex cannot be relied on. Additionally, cremasteric reflexes are absent in 30% of males with normal testicles.^1^ Isolated pain along the upper pole of the testicle or epididymis has been reported to occur in 18.7% of patients with TT and 40.8% of patients with torsion of the testicular appendage.^1^ A transverse testicular lie has been reported in 17% to 83% of TT cases, while a vertical lie has been observed in up to 54% of cases of TT.^1^ Lastly, testicular retraction (high-riding testicle) is only present in 33%–80% of TT cases.^1^

### Dr. Couperus

A scrotal ultrasound, the “gold standard” test, can be very helpful, although it is not foolproof. Lawyers will argue that “one simple and available test” could have been ordered and made the diagnosis. However, upon review of cases involved in litigation, we found that obtaining an ultrasound did not correlate with a more successful defense.^(2,6)^ This is because a scrotal ultrasound can be misread as normal by radiologists.^(2,6)^ In general, high resolution ultrasonography has a sensitivity of 96% but is not perfect.^6^ If a negative ultrasound is reported, in the situation that a high clinical suspicion remains, a urologist should be consulted. Involving a consultant has historically created a very defensible position.^6^

The time window for possible salvage and survival of a torsed testicle is commonly thought to be 6–8 hours.^7^ Recently, a review of 30 articles, with over 2,116 patients included, looked at outcomes related to time of torsion. When reported in six-hour intervals (1,283 patients), survival at 0–6 hours was 97.2%; 7–12 hours, 79.3%; 13–18 hours, 61.3%; 19–24 hours, 42.5%; 25–48 hours, 24.4%; and greater than 48 hours, 7.4%. Cumulative testicular survival data based on reporting for all three groups of patients were as follows: testicular salvage in the first 12 hours is 90.4%; from 13–24 hours survival is 54.0%; and beyond 24 hours survival is 18.1%. Vigilant urgency is prudent irrespective of the time that symptoms have been present when TT is a consideration.^7^

## MEDICOLEGAL ISSUES

### Dr. Pfaff

A review of jury verdict reports in cases of TT was done to identify causes of litigation and factors contributing to verdicts or settlements.^1^ This review examined 52 pertinent case outcomes in which 51% resulted in favor of the physician and 49% in indemnity payment. EPs were the most commonly sued medical providers (48% of defendants), followed by urologists at 23%, and were significantly more likely to make indemnity payments than urologists. The majority of malpractice claims were failure of diagnosis (96%). Misdiagnosis of epididymitis was noted in 27 cases (65%).^8^ A retrospective review of TT malpractice cases from 1985 to 2015 reported similar findings in 53 relevant cases, 88% with testicular loss.^2^ Again, EPs were the most common type of provider sued (35%) followed by family physicians (17%), and urologists (13%). However, specialty was not shown to be associated with successful defense. Most claims for malpractice included missed diagnosis and negligence (98%). Half of providers diagnosed patients with epididymitis on first presentation (52%). Atypical presentation (31% with abdominal pain only) and failure to complete a testicular exam was associated with 19% of TT malpractice cases.^8^ False negative ultrasound findings were common among these cases. When a radiologist misreads the testicular ultrasound, often the radiologist pays out less than the EP, or the EP pays out alone. The thought process is that the EP had the ability to make a “clinical correlation” that the radiologist didn’t.^2^

### Dr. Moore

The sudden onset of severe, unrelenting testicular pain is typically held to be diagnostic of TT. This is not the case, however, for a small but significant number of patients with a torsed testicle. The little-recognized fact that TT patients can present with minimal or no pain has proven to be a medicolegal pitfall for EPs.^2^ A subset of TT patients reports resolution of their initial severe pain followed by variable periods of hours to days of reduced or absent pain. Other patients report only mild pain described as gradual in onset. These “pain honeymoons” may be partially responsible for poor clinical outcomes because of delayed initial presentations or less-than-timely returns for secondary evaluation. The pain relief experienced by some patients with TT has been likened to an extremity paresthesia that develops after prolonged nerve compression. The pain again begins to worsen, and secondary scrotal inflammation and pain occur as inflammatory factors increase with infarction of the testicle.

A recent article highlights seven cases of TT and raises a serious liability concern. In all of these patients, there was a period of freedom from pain, or much decreased pain after the initial onset of symptoms (“pain honeymoon”). The diagnosis would be very easy to miss in this clinical scenario. The mechanism is thought to be one of compression of the nerves as they travel in the spermatic the cord with resultant paresthesia and anesthesia.^7^

## CONCLUSION

Missed TT is a frequent source of successful litigation against EPs. There are many traditional paradigms in the areas of history, physical exam, imaging studies, and clinical course that can lead to diagnostic failure. Given these clinical uncertainties and high risk for testicle loss, EPs should routinely document a scrotal exam for young males with lower abdominal pain, have a low threshold for ultrasound imaging with any reasonable suspicion, and use a liberal threshold for urological consultation, if available.

## TAKE HOME POINTS

1. Successful litigation for testicular torsion often occurs due to failure to do a genital exam in patients with abdominal pain.
2. Successful litigation for TT often occurs by a failure of the radiologist to notice pathology on scrotal ultrasound. Nevertheless, the EP is held responsible. A urologist should be involved when there is high clinical suspicion for TT in the face of a “negative” ultrasound.
3. Testicular salvage after 24 hours of torsion is still 18%, and physicians should aggressively pursue the diagnosis even in a delayed presentation.
4. The overwhelming majority of malpractice claims were failure of diagnosis, and 2/3 of these cases were diagnosed as epididymitis.
5. Recent reports described cases of “honeymoon” absence of pain in TT. The improvement of testicular pain or its absence after initial onset should *not* reassure the provider that the diagnosis is not likely.

Documented patient informed consent and/or Institutional Review Board approval has been obtained and filed for publication of this case report.
